# Exploring the Need for Mobile Application in Stroke Management by Informal Caregivers: A Qualitative Study

**DOI:** 10.3390/ijerph191912959

**Published:** 2022-10-10

**Authors:** Muhammad Iqbal Haji Mukhti, Mohd Ismail Ibrahim, Tengku Alina Tengku Ismail, Iliatha Papachristou Nadal, Sureshkumar Kamalakannan, Sanjay Kinra, Jafri Malin Abdullah, Kamarul Imran Musa

**Affiliations:** 1 Department of Community Medicine, School of Medical Sciences, Health Campus, Universiti Sains Malaysia, Kubang Kerian 16150, Kelantan, Malaysia; 2 Division of Care in Long Term Conditions, King’s College London, London SE1 8WA, UK; 3 Department of Non-Communicable Disease Epidemiology, London School of Hygiene & Tropical Medicine, Faculty of Epidemiology and Population Health, Keppel Street, London WC1E 7HT, UK; 4 Department of Social Work, Education and Wellbeing, Faculty of Health and Life Sciences, Northumbria University, Newcastle NE7 7XA, UK; 5 Department of Neurosciences, School of Medical Sciences, Health Campus, Universiti Sains Malaysia, Kubang Kerian 16150, Kelantan, Malaysia; 6 Brain and Behaviour Cluster, School of Medical Sciences, Health Campus, Universiti Sains Malaysia, Kubang Kerian 16150, Kelantan, Malaysia; 7 Department of Neurosciences & Brain and Behaviour Cluster, Hospital Universiti Sains Malaysia, Health Campus, Universiti Sains Malaysia, Kubang Kerian 16150, Kelantan, Malaysia

**Keywords:** mobile application, stroke management, informal caregivers, healthcare digitalization, rehabilitation

## Abstract

Background: Mobile health (mHealth) has been considered as a prominent concept in digital health and is widely used and easily accessible. Periodic follow-up visits, previously planned procedures, and rehabilitation services for stroke survivors have been cut down during the recent COVID-19 pandemic. Therefore, in this qualitative study we aimed to explore the need for a mobile application in stroke management by informal caregivers. Methods: A phenomenological qualitative study was conducted from November 2020 to June 2021. Thirteen respondents were recruited from two public rehabilitation centers in Kota Bharu, Kelantan, Malaysia. In-depth interviews were conducted. A comprehensive representation of perspectives from the respondents was achieved through purposive sampling. The interviews were conducted in the Kelantanese dialect, recorded, transcribed, and analyzed by using thematic analysis. Results: Thirteen participants were involved in the interviews. All of them agreed with the need for a mobile application in stroke management. They believed the future stroke application will help them to seek information, continuous stroke home care, and help in the welfare of caregivers and stroke patients. Conclusions: The current study revealed two themes with respective subthemes that were identified, namely, self-seeking for information and reasons for using a stroke mobile application in the future. This application helps in reducing healthcare costs, enhancing the rehabilitation process, facilitating patient engagement in decision making, and the continuous monitoring of patient health.

## 1. Introduction

Innovation in the healthcare sector has evolved over time since decades ago. In the early phase of healthcare digitalization, telemedicine was first introduced as healing at a distance [1,2]. This approach is perceived in a broader scope by incorporating nurses, pharmacists, and public health management [3,4]. Later, the technology evolved again and electronic health (eHealth) was introduced. It is described as a field of medical informatics, public health, and business related to healthcare service delivery that is enhanced through the Internet and other technologies [5,6]. This involves private and non-profit partnerships, such as with telecommunication agencies, in order to keep services cost-effective and to effectively deliver public healthcare services by making use of eHealth services extensively, to enhance the existing healthcare service delivery and strengthen integrated and people-centered healthcare services in order to contribute to health equity and gender equality, and consequently improve the population’s health [7,8]. To ensure that this aspiration is focused, objective, and standardized, the World Health Organization (WHO) emphasized the agenda of digital health through the Global Strategy on Digital Health 2020–2025, which is a comprehensive and sturdy initiative accommodating financial, organizational, human, and technological resources to obtain high-quality and widely accessible healthcare services [9], thus promoting health and wellbeing at every segment of individuals and populations, at all ages.

Digital health encompasses a wide range of technologies, including mobile health (mHealth), telehealth and telemedicine, wearable devices, health information technology (IT), and personalized medicine, which catalyze the effectiveness of healthcare delivery [10]. The widespread use of mobile phones and personal digital assistants led to the growth of mHealth as a prominent concept in digital health which is widely used, easily accessible, and closely related to the genomic data and information of individuals that provide public health information and services, allowing professionals to monitor patients and thus enhancing the quality of healthcare for a better health outcome [11,12,13]. Digitalization transforms healthcare and promotes teams to work in a way that is integrated, flexible and interoperable, and enabled digitally cared environment leverages [14]. Digital health benefits personnel to make use of technologies, empowering patients in terms of self-management and caregivers’ involvement, thus improving patients’ wellbeing [15]. Furthermore, Malaysia continues to have a scarcity of teleconsultation services in public primary healthcare clinics, with low usage among medical professionals [16]. In comparison to other Southeast Asian countries, there appears to be room for improvement in telemedicine and telehealth guidelines [17].

Following the World Health Organization’s (WHO) declaration of the COVID-19 pandemic [18], numerous efforts were made to reorganize medical assistance for a variety of diseases, including stroke. To maintain care management, new pre-hospital and in-hospital acute stroke pathways were proposed [19]. During the pandemic, the treatment of many stroke patients became delayed. One of the reasons for this was apprehension about in-hospital infection. Second, health authorities, the media, and doctors may advise patients with mild symptoms to remain at home. Furthermore, caregivers failed to recognize stroke as an emergency and paid no attention to the acute appearance of minor symptoms that should be treated immediately, and the COVID-19 pandemic caused a lot of confusion for caregivers in managing stroke patients at home [20].

Many stroke units were closed or converted into COVID-19-positive wards in order to reallocate stroke physicians and nurses to the care of COVID-19 patients [20,21,22]. Owing to the need to allocate wards to COVID-19 acute management, post-stroke rehabilitation pathways were also centralized, and rehabilitation care and services were divided into COVID-19-free and COVID-19-positive areas to ensure adequate measures for patient isolation [19]. Periodic follow-up visits, previously planned procedures, and rehabilitation services for stroke survivors were reduced globally as a result of COVID-19 control measures [20,21]. This prompted the question of how to care for patients who require regular consultations and rehabilitation assistance. To overcome these constraints, some patients were contacted by phone, and telemedicine platforms were put in place to allow patient visits, avoiding direct contact with operators and waiting rooms [23,24,25].

Despite all the crucial difficulties based on the justifications above, there are still a small number of studies that focused on digitalization access and the use, importance, and benefits of a mobile device application for healthcare purposes. This was primarily for stroke care management by informal caregivers, particularly in the local context. Thus, our goal in this study is to investigate by a qualitative approach if a mobile application is necessary for informal caregivers to manage stroke patients.

## 2. Materials and Methods

### 2.1. Study Design

A qualitative study using an exploratory phenomenological approach was conducted to investigate how informal caregivers perceive and interpret the use of stroke mobile applications in the care of stroke patients.

### 2.2. Study Participants

There were two main public rehabilitation centers involved, namely, Pusat Pemulihan & Rehabilitasi Hospital Raja Perempuan Zainab II (HRPZ II) and Hospital Universiti Sains Malaysia (Hospital USM) in Kota Bharu, Kelantan. The study was conducted from November 2020 to June 2021. Families taking care of stroke victims who registered at Hospital USM or HRPZ II and met the following requirements made up the source population. The caregiver must be the stroke patient’s spouse, another family member, or a relative who is at least 18 years old and open to sharing their experiences.

The criteria used to choose the stroke patients were outlined in the Malaysia Clinical Practice Guidelines, which are primarily atherothromboembolism, intracranial small vessel disease, cardiogenic embolism, and others [26]. Stroke presentation caused by transient ischemic attacks, tumors or malignancies, spinal cord injuries, and motor vehicle accidents were excluded. Apparently, the sample size for qualitative research is not determined by a scientific method [27], but a study showed that data in qualitative research is considered sufficient once it is saturated [28]. Nevertheless, for practical purposes, a group of 12 to 26 respondents is adequate [29]. Hence, a total of 13 respondents participated until data attained saturation, which is a requirement for data sufficiency [30]. Two of the participants came from HRPZ II (with additional three participants involved in pilot interview), and the rest were from Hospital USM.

### 2.3. Data Collection

The expert team members prepared and validated an interview guide before the participant selection procedure. They consist of three public health clinicians, one rehabilitative medicine specialist, and two qualitative experts from London School of Hygiene & Tropical Medicine. To ensure that a variety of participants were selected, the maximum variation or heterogeneous purposive sampling technique [31] was applied to the sample of registered stroke patients diagnosed by neurosurgeons or neuromedicals and physiotherapists at either of the two listed centers or both. As stroke patients and their caregivers attended the appointment, the clinical teams first identified the appropriate participants. A researcher was then contacted by the team to obtain consent from the patient and caregivers in a similar setting to avoid any dropout potentiality if the meeting was reset to another day. The study process was thoroughly explained to the participants, who were also made aware of their right to discontinue at any moment. Written consent was obtained before the interviews and signed by the participants. The in-depth interviews were conducted and recorded using a digital voice recorder with permission. Revisions on the adjustments and enhancements to the interview guide were made after the first two pilot interview sessions, conducted before the major study. Each interview lasted between 30 and 45 min, and if necessary, a revision was made for any session to obtain more accurate and significant information. All of the data obtained were eventually included in the data analysis.

Overall, three main questions were asked: “How do you seek information about managing a stroke patient?”, “How likely are the challenges of your stroke caregiving be the key factors driving demand for a stroke mobile application?”, and “From your viewpoint, to what extend can a stroke mobile application be of help to you as a caregiver and a stroke patient?” Appendix A (Table A1) shows a detailed breakdown of the main and probing questions. The interview sessions were conducted in Kelantanese, a native Malay local dialect. According to O’Reilly and Parker, the data collection ceased after the data achieved saturation, meaning that no new themes, coding, or data were given in the following sequential samples [32].

### 2.4. Data Analysis

There were 13 interviews conducted in total, and the last two sessions showed that no new data were generated, indicating that the data were sufficient [33]. This study used a six-phase thematic analysis method, as described by Braun and Clarke: (i) familiarizing oneself with the data, (ii) generating initial codes, (iii) looking for themes, (iv) reviewing themes, (v) defining and labeling themes, and (vi) writing the report [34]. The first author transcribed verbatim the recorded interviews, and the other authors double-checked the transcripts. The complete interviews were transcribed into Kelantanese Malay, and the first coding was done after reading and rereading the first three interviews numerous times to become comfortable with and better understand the context of the material provided by the subjects. The first author then identified the information that had been taken from each interview session and independently examined it to create the correct code. This was accomplished by creating comments and codes for the data extraction margins. Next, all the authors discussed and examined the data extraction codes’ results until a consensus was reached. The other interviews were conducted using a similar procedure. To review, discuss, and settle on the coding, all the authors met numerous times. After that, the codes were sorted into likely subthemes and finally into emergent themes. A thematic map was made on the basis of the retrieved codes and subthemes in order for themes to emerge. The sense of significance, relevance, and different relationships among the topics were specifically defined and designated to convey the meaning and were then further discussed and examined by the authors until an agreement was reached. The best analytical output was the final report of the data extraction linked to the research question and study topic appropriate to the literature.

### 2.5. Ethical Consideration

The participants were notified in advance regarding their voluntary participation, and they were free to leave the interview at any time, withdraw, or choose not to respond without incurring any consequences, including being penalized. The recordings were labeled with specific identification numbers and kept confidential and secure. Ethical approval was granted by the Medical Research and Ethics Committee (MREC), Ministry of Health Malaysia [code: NMRR-20-351-53369 (IIR)] and the Human Research Ethics Committee, USM [code: USM/JEPeM/20010031].

## 3. Results

### 3.1. Characteristics of Respondents

The traits of informal caregivers and their connections to stroke survivors are shown in Table 1. The 13 participants in this study came from a variety of socioeconomic backgrounds, with the majority being Malay. There were also two respondents from different ethnic groups: Chinese and Indian.

Two themes with listed subthemes emerged on the need for a mobile application in managing stroke patients by informal caregivers, as shown in Table 2. The initial codes for each subtheme are detailed in Appendix B (Table A2).

### 3.2. Theme 1: Self-Seeking Information

Given that managing a stroke patient takes a fair amount of time, caregivers must have sufficient knowledge and skills in this area. Most respondents said that as a stroke patient’s caregiver, they felt that information and knowledge should not only be limited to managing a stroke patient’s daily activities but should also more broadly encompass welfare and other issues that frequently presented problems for both patients and caregivers.

#### 3.2.1. Subtheme: Obtaining Missing Information

Not all information and knowledge were available to caregivers and stroke patients, despite the significant role rehabilitation clinics played in providing training and feedback for the healing of stroke patients. As a result, it led to extra efforts from the caregivers, who took the initiative to look for a variety of available approaches to equip themselves with knowledge and information on daily management and issues related to the welfare of stroke patients, in addition to what has been provided by rehabilitation centers. Inadvertently, this circumstance showed that the information available was still insufficient and that part of it was within the purview of treatment facilities. According to one respondent, the availability of a mobile application for stroke patients may be useful as an extra resource for any information offered by rehabilitation facilities about the care of stroke patients, which is still lacking.

*“… even here in the hospital, we can see how the stroke management is done… so I learned… but I couldn’t remember it most of the time and it was always lacking… I need some more feedback… So, with this suggested mobile application, things will improve and become much easier… If I have any doubts, I can use it as a reference…”*

[R7, 35, wife]

The lack of information was not limited to physiotherapy and the management of stroke patients in daily life, but also included information on welfare aid. According to one respondent, non-governmental organizations (NGOs) and rehabilitation centers should work together to provide welfare aids to stroke patients. After all, information about NGOs that provide welfare assistance to stroke patients has been widely disseminated. There appears to be no problem with the platforms in providing welfare aid, but this is more likely due to a lack of information and knowledge about how to apply for welfare aid. Caregivers were thus concerned with developing a stroke mobile application to meet this purpose, while also accommodating the shortcomings mentioned above.

*“… as an example, consider an NGO like YOKUK… I believe that NGOs and hospitals can work together to help stroke patients… I mean, proactive action is being taken, primarily to involve those responsible… It might be useful… But at first, any information provided went unnoticed… we rarely have ideas for things like collaboration between two organizations… so, if there is a mobile app for this, it could be useful…”*

[R11, 66, husband]

Additionally, with the welfare issue specifically brought up, both intra-agency and inter-agency communication issues were notably prevalent as they impacted everyone from top management to lower-level employees. One respondent drew attention to the mechanism’s inability to effectively disseminate information about any enforced policies. Networking and connection greatly impacted the communities, particularly those connected to the service given, as a result of issues with policy ambiguity among employees, ineffective task delegation in service implementation, and suboptimal use of digital technological innovation. Consequently, it is envisaged that the widespread use of a stroke mobile application will contribute to improving the information distribution system for the benefit of the general population.

*“… sometimes a department’s policy is good, but when it comes to implementation, it’s simply unworkable… possibly due to issues with the information delivery mechanism at a lower level… the lack of communication between policy implementers obscures them, compromising the effectiveness of service delivery… Worse, unimproved IT and networks rendered information on existing services unavailable to the public…”*

[R11, 66, husband]

#### 3.2.2. Subtheme: Internet Accessibility for Feasible Caregiving

Stroke sufferers might receive information that goes beyond what is provided in rehabilitation facilities by gaining knowledge online. Caregivers who lacked experience and were unfamiliar with the treatment of stroke patients were more likely to use this technique. One respondent acknowledged that the use of a stroke mobile application can facilitate the process of therapy, managing care, and medication intake through Internet browsing:

*“… previously, if I wanted to know how to care for a stroke patient, especially how to help him perform physiotherapy or any information on medications that were prescribed, I would search for it on Google… because I had no prior experience providing care to stroke patients… So, if a stroke mobile application is available, I can use it… it is pleasant for me.…”*

[R3, 44, wife]

Nevertheless, caregivers often search the Internet for expertise and information on managing and lowering stress related to the disabilities of the patient because stroke patients may have many disabilities, such as slurred speech and poor gait. They value this because it will at least lighten their load. According to one respondent, it is anticipated that this service will be provided with the help of a mobile application for patients with stroke:

*“… I also learned to practice her physiotherapy by watching YouTube and searching information on Google… if a stroke mobile application is available, she can learn to practice other relevant exercises as well, particularly to strengthen her memory, in addition to routine physical exercises…”*

[R11, 66, husband]

More importantly, caregivers’ requests for knowledge and information were not limited to a single website. For comparison, they acquired information from several sources both locally and globally. To make it easier for people to understand and adjust to the updated physiotherapy technique, one respondent expressed the importance of combining all the information from all sources into one stroke mobile application. However, this information must be appropriately adjusted following local norms.

*“… indeed, I learn about physiotherapy practice from YouTube and Internet searches… some are similar to what has been done in this rehabilitation center, but others are not and are only performed abroad… If there is a stroke mobile app, all of the techniques can be combined to meet local needs… it makes my life easier, and I’d like to keep doing it…”*

[R5, 29, daughter]

### 3.3. Theme 2: Reasons for Using a Stroke Mobile Application in the Future

Caregiving for a stroke patient is associated with a high workload, as is maintaining continuous care daily for an extended period. The longer it took for stroke patients to recover, the longer caregivers were exposed to heavy and tiring caregiving tasks, unintentionally exacerbating their physical and psychological distress. To overcome this ultimate impact, a stroke mobile application was seen as a solution to the numerous problems encountered by caregivers while managing stroke patients in daily life.

Evidently, all respondents interviewed were likely to demonstrate favorable and constructive feedback on the use of a stroke mobile application that serves as a significant tool to facilitate the management of stroke patients, particularly during pandemic circumstances.

#### 3.3.1. Subtheme: Causal Factors Prompting the Use of the Application

Respondents provided several reasons to justify the use of a stroke mobile application. According to one respondent, the presence of a mobile application could be useful when she was unavailable due to her personal responsibilities. Her mother is semi-dependent on daily activities and would most likely be able to use the application without too many difficulties.

*“… I’m 18 years old and I’m still in school… If I have time, I’ll assist her… She can read and type messages on WhatsApp on her own… aside from cooking and light household chores… If there is a stroke mobile application, I believe it would be beneficial to her…”*

[R9, 18, daughter]

Furthermore, one respondent stated that she did not have time to search for knowledge and information on the Internet or chat on social media owing to the time constraints of caring for a stroke patient. She had to rely entirely on the information provided by the rehabilitation center during physiotherapy sessions at the time. Because information can be obtained from a single source, the presence of a stroke mobile application is expected to aid in the management of stroke patients.

*“… I didn’t even look at the Internet, websites, or social media… I didn’t have much time because I was too busy managing him on a daily basis… As a result, the majority of the stroke-related information I learned while attending appointments here… It would be ideal if a stroke mobile application could be developed… It will assist me in managing my father’s stroke condition, especially when all the information I require is available on a single platform…”*

[R1, 24, daughter]

Additionally, based on the comments from respondents, a person who has been dealing with a persistent disability for an extended length of time is likely to develop physical and mental exhaustion. When the illness first started, they regularly adopted active lifestyle habits, but with time that started to decline. Therefore, respondents hope to increase motivation by sharing experiences and success stories through a stroke mobile application.

*“… when he first had a stroke in 2013, he would frequently go for massage… every morning, he jogged around the house… now, perhaps because of the disease’s long-term effects, he doesn’t want to do it… If only there was a mobile app that could help him gain more motivation…”*

[R6, 25, son]

*“… just that she’s resting more and not exercising… I really have to remind her to do anything; she needs to do regular exercise on a regular basis, just like she used to… Perhaps she is demotivated because she has been at home for a long time and feels disabled… It would be fantastic if there was a mobile application that could help to support and encourage her…”*

[R2, 43, husband]

#### 3.3.2. Subtheme: Facilitating Home Physiotherapy

Some of the problems that frequently impede home physiotherapy can be resolved by using a stroke mobile application. Some caregivers expressed that doing physiotherapy at home should be simple. It should be conducted for a long time as a regular routine. However, patients can easily lose motivation if home physiotherapy is difficult because they have already undergone a protracted rehabilitation process. Therefore, a stroke mobile application was anticipated to provide an affordable and simple guide for completing at-home physiotherapy.

*“… we only have balls and other simple tools to use at home… neither pedal for cycling… just me and my effort to teach her to walk, help her to hold a bed, grasp hands, and support her… do what is easy… if any mobile application can help us get cheap tools, modify the existing tool, it would be great…”*

[R13, 32, son]

A similar respondent stated that a stroke mobile application should also include references to the appropriate physiotherapy procedures. This is crucial to prevent stroke patients from feeling any discomfort or pain and stiffness in their muscles and joints as a result of improper physiotherapy. Indirectly, it might impact the continuity of therapy at home, hastening the recovery.

*“… sometimes, one may not know how to do home physiotherapy, how to manage a stroke patient… one never knows if they are hurt, uncomfortable, or dislike… moreover, if they were managed incorrectly, it might cause muscle and joint stiffness… what a pity for them… that’s why with a stroke mobile application, it will show the right way of performing the exercises…”*

[R13, 32, son]

The use of a stroke mobile application, according to a different respondent, can serve as a reminder by providing both written and visual physiotherapy instructions. With this benefit, it may also be utilized as a guide to remember the physiotherapy exercises carried out at rehabilitation facilities after returning home. This can guarantee that a chronic patient receives continuous care.

*“… sometimes we can’t remember everything we learned at the rehab center when we get home… unless we write it down and refer to pictures taken during the session… so, if we have a mobile app that uploads the procedure step by step, we can go through it again and again… If only it were possible…”*

[R11, 66, husband]

#### 3.3.3. Subtheme: Welfare as a Necessity

Nonetheless, it is widely acknowledged that caring for stroke patients necessitates a consistent and lengthy rehabilitation process to improve their recovery. As a result, various aspects of welfare must be taken seriously to truly ensure the holistic continuity of care for stroke patients. These include financial assistance, physiotherapy equipment, supplemental feedings, and a variety of other services for stroke patients. All these factors necessitate the participation and cooperation of numerous authorities. Typically, caregivers must locate each of the respective agencies on their own. It takes time to find a solution, fill out a form, and then wait for approval. As a result, respondents suggested that a stroke mobile application be developed to solve this problem by providing a service center for stroke care.

*“… I believe it would be beneficial if any link to the responsible agencies could be attached via a stroke mobile application… It would be nice, and it would make my caregiving task easier…”*

[R6, 25, son]

*“… more importantly, we would quickly notice any type of assistance with the mobile application… sometimes, those who qualify but do not receive supplementary milk or physiotherapy tools such as a wheelchair… they may have chances to request by using this application…”*

[R13, 32, son]

### 3.4. Recommendation for the Content of Development of Stroke Mobile Application

Based on the current finding we could summarize the content of the application as suggested by the respondents:

No. Recommendation of content

1. General knowledge and information on stroke caregiving on daily routines such as eating, bathing, walking, sitting, standing, etc.
2. Knowledge and information on contextual stroke caregiving that appropriate with current COVID-19 pandemic.
3. Motivational spirit to keep consistent on rehabilitation process.
4. General knowledge and information to practice home physiotherapy.
5. Technical skill in performing home physiotherapy in the easiest way, appropriate and correctly.
6. Financial and welfare support for sustainability of stroke care.

## 4. Discussion

The “self-seeking information” theme was reinforced throughout this study’s findings and was further described by two subthemes, namely “obtaining missing information” and “Internet accessibility for feasible caregiving.” This demonstrated that, despite the widespread use of the Internet, many caregivers continued to seek information and knowledge in managing stroke patients, which was lacking, incomplete, and difficult to obtain from traditional sources, primarily rehabilitation centers. Previous research found that a lack of specific knowledge and information on the illness and the patient’s issues and needs were indeed common issues for family caregivers [37,38,39]. This includes social support, such as the availability of welfare aids, which is commonly associated with a lack of long-term support or stroke aftercare [40] and a perceived lack of attention regarding patients’ social recovery [41,42,43], as revealed by this study. The higher the level of social support, the faster the rate of recovery, and the greater improvement in the functioning of stroke survivors when compared to those who received inadequate care, which typically resulted in poor outcomes [44].

The current fragmented approach to stroke care is one of the challenges in healthcare services. This leads to poor management coordination and rehabilitation, and the situation worsens when dealing with rural populations [45,46,47]. As a result, the Institute of Medicine (IOM) reported that the challenges have resulted in suboptimal treatment and inefficient use of healthcare services. To address this, it is recommended that coordinated care systems be established to integrate evidence-based preventive and treatment care services [48,49,50]. Having a mobile application for stroke care is one example. However, difficulties are also observed from the viewpoint of caregivers and stroke patients through the “causal factors prompting the use of the application” subtheme that developed from the second theme. This subtheme refers to the lack of time and attention from caregivers on stroke caregiving owing to their other commitments, which in turn interferes with the process of physiotherapy, and demotivates patients with physically and intellectually impaired lives. Healthcare and the public sector, which are frequently considered to be the least digitally mature industries with a correspondingly lower success rate in digital transformation, are also focused on during the process of digitalization and its transformation, in addition to businesses, institutions, and industries that are already digitally advanced [51]. Nevertheless, the digitalization shift in health has had an impact on the public, including patients and caregivers, who are increasingly connected to a wide range of devices and social networks [52]. As a result, individuals are easily exposed to information technology that is fast developing and spreading. Therefore, the availability of sophisticated and frequently updated technologies would aid in making knowledge and information conveniently accessible over the Internet for each individual in any situation, at any time, and everywhere. Digital accessibility encourages people to live in a world of endless information and communication [53]. Without a doubt, this demonstrated the varied ways in which technology can influence society. This is especially true for stroke victims and their caregivers in the context of healthcare.

To better understand stroke mobile applications, a systematic review of 843 out of over 30,000 medical apps in the US Apple iTunes store, which met the inclusion criteria, was conducted between August 2013 and January 2016 [54]. Of the 843 apps, 8.7% (74 applications) were specifically made for stroke survivors/caregivers. Only 9% were designed for numerous functions, and 57% were expensive to download. These included assistance with language/speech therapy (37%), communication with aphasic patients (25%), stroke risk assessment (14%), assistance in identifying an acute stroke (10%), atrial fibrillation detection (4%), pointing patients in the right direction to an emergency room (4%), physical rehabilitation (4%), pointing patients in the right direction to the closest certified stroke center (2%), and visual attention therapy (2%). While only 15% of these applications (out of 769 in total) were created for numerous purposes, 91.3% were created for purposes other than stroke. The majority (68%) of the content addressed modifiable risk factors for stroke, while the majority of the app focuses on a single risk factor. These risk factors included obesity/weight loss (46%), diabetes (34%), hypertension (26%), smoking (10%), dyslipidemia (6%), and atrial fibrillation (1%). The remaining 32% provided information on how to communicate with an aphasic patient, access language/speech therapy, find the closest hospital or emergency room, an improve the quality of care, physical therapy, hearing and balance impairments, memory deficits, and vision impairments that stroke survivors and caregivers can use.

The current findings revealed that the respondents understood that the future stroke mobile application is a multifunctional hub that allows access to accurate and authentic sources of information, in addition to being a time-saving application. This is in line with recent research [55,56,57] that emphasized the advantages of using multipurpose mobile applications, especially in terms of time saving [58,59]. To create a paradigm shift in stroke healthcare toward achieving a proactive involvement in patient self-care practices and improving patient engagement instead of being a passive recipient of self-healthcare, the application should be able to deliver the appropriate and intelligible information according to the local environment [60,61]. Therefore, by improving physical and mental conditions, it will be possible to minimize hospital admission and stays, cut healthcare costs and malpractice claims [62], and ultimately improve the quality of life of patients [63]. Parallel to this, according to the IOM, the advancement of healthcare technology over many years has ushered in the current digital health era and has continued to move quickly toward a patient-centric approach that is also safe, effective, timely, efficient, and equitable in terms of the enhancement of healthcare service quality. This principle focuses on responding to and respecting patients’ preferences, needs, and values while ensuring that their values are considered when making healthcare decisions [48].

The “facilitating home physiotherapy” and “welfare as a necessity” subthemes in the second theme outlined the role of the stroke mobile application in ensuring the continuity and consistency of home physiotherapy that is also flexibly, practically, and safely performed, especially during the pandemic. It helps in the continuity of care, aside from establishing motivational and psychological support for chronic stroke patients and caregivers and strengthening social support, such as providing financial and welfare assistance for the basic needs of both patients and caregivers.

### Limitations of This Study

Owing to time, logistical, and technical limitations brought on by the pandemic, the caregivers in this study were only interviewed once; as a result, it is possible that they did not provide all the information requested during the session. Furthermore, the interviewer’s use of the local dialect during the interview raised the possibility that the message was misunderstood or interpreted differently. Through the translation of the local dialect, data transcription, or the entire process of data analysis, a similar issue was raised in entire process. The findings of this study, which were restricted to the two main rehabilitation facilities in Kota Bharu, Kelantan, and concentrated mostly on informal caregivers of stroke patients, may not generalize to other contexts in Malaysia or elsewhere. Only those caregivers who nearly share the same demographic, social, cultural, religious, and ethnic characteristics as well as similar settings and rehabilitation center protocols qualify for the exception.

## 5. Conclusions

According to our findings, all of the respondents interviewed believed that a stroke mobile application can help improve stroke caregiving management. It is widely regarded not only as a mediating tool for improving a stroke patient’s quality of life but also for promoting the welfare of caregivers by assisting them in reducing the burdens associated with managing stroke patients on a daily basis. Incorporating technology reduces the barriers and limitations within a healthcare system, thereby increasing the efficiency and effectiveness of services. In a nutshell, coordinating digital health in promoting health education, eliminating barriers to specific care, enhancing the rehabilitation process, facilitating patient engagement in decision-making, improving communication between providers and patients, and continuous monitoring of a patient’s clinical status may improve the stroke healthcare system in the long run.

## Figures and Tables

**Table 1:** Characteristics of informal caregivers and relationship to the stroke survivor.

No.	Sex	Age	Educational Level ^a^	Employment Status ^b^	Household Income/Month (RM) ^c^	Relationship to the Stroke Survivor
1	F	24	Intermediate	Unemployed	2500	Daughter
2	M	43	High	Employed	13,000	Husband
3	F	44	High	Employed	11,500	Wife
4	F	37	High	Unemployed	6000	Wife
5	F	29	High	Self-employed	12,000	Daughter
6	M	25	Intermediate	Employed	8000	Son
7	F	35	High	Unemployed	8400	Wife
8	M	59	Intermediate	Employed	7500	Father
9	F	18	Intermediate	Unemployed	3000	Daughter
10	F	54	Intermediate	Unemployed	5500	Wife
11	M	66	High	Retired	8500	Husband
12	F	57	Intermediate	Unemployed	2800	Wife
13	M	32	High	Unemployed	5000	Son

^a^ Low: elementary school or low vocational education. Intermediate: secondary school or intermediate vocational education. High: higher vocational education or university education [35]. ^b^ the employment status was categorized on the basis of Rahman et al. [36]. ^c^ 1 USD = RM 4.50. F = female, M = male.

**Table 2:** Themes and subthemes regarding the need for a mobile application for managing stroke patients by informal caregivers.

Themes	Subthemes
Self-seeking information	Obtaining missing information
	Internet accessibility for feasible caregiving
Reasons for using a stroke mobile application in the future	Causal factors prompting the use of the application
	Facilitating home physiotherapy
	Welfare as a necessity

## Data Availability

The data presented in this study are available upon request from the corresponding author. Due to privacy concerns, the data are not publicly available.

## Appendix A. Main and Probing Questions for the Guided Interview

**Table A1:** Examples of main and probing questions.

No.	Directions		Questions
1.	Experience in seeking information	Main	How do you seek information about managing a stroke patient?
		Probing	What information do you frequently seek out, which you believe will be essential for you as a caregiver to help you with the daily task of caring for a stroke patient?
2.	Reasons for using a mobile application in the future	Main	How likely are the challenges of your stroke caregiving be the key factors driving demand for a stroke mobile application?
		Probing	What challenges did you face while taking care of a stroke patient?
3.	Content of a stroke mobile application	Main	From your point of view, to what extend can a stroke mobile application be of help to you as a caregiver and a stroke patient?
		Probing	What kind of cellphone do you use daily and how familiar are you with the applications in your cellphone?
			What is your opinion if a mobile application that may help you manage a stroke patient existed on a cellphone?
			From your experiences in managing a stroke patient, what information should be included in a stroke mobile application that will help you as a caregiver?
			How can a stroke mobile application possibly affect your daily care and management of a stroke patient?

## Appendix B. List of Initial Codes

**Table A2:** Themes and subthemes regarding the need for a mobile application for managing stroke patients by informal caregivers.

Themes	Subthemes	Initial Codes
Self-seeking information	Obtaining missing information	Self-initiatives given the insufficient information provided by rehabilitation centers
		Insufficient information on the provision of welfare aids particularly by NGOs
		Inefficient information delivery mechanism with ineffective intra- and interagency communication
	Internet accessibility for feasible caregiving	Online information helps inexperienced stroke caregivers
		Online information guided caregivers in managing different patients with different stroke disabilities
		Online information provides knowledge on caregiving according to the local context
Reasons for using a stroke mobile application in the future	Causal factors prompting the use of the application	Lacking time for caregiving due to other commitments
		Too busy providing care to find the time to seek information and knowledge
		Inappropriate caregiving information especially during the current COVID-19 pandemic
		Low enthusiasm due to long duration and persistent disabilities
		No driving force or self-motivation to practice home physiotherapy
	Facilitating home physiotherapy	Help to overcome financial constraints while practicing home physiotherapy
		Help to simplify home physiotherapy in the easiest way
		Provide information on appropriate and correct home physiotherapy
		Serve as recall references in performing home physiotherapy
